# Revealing topics and their evolution in biomedical literature using Bio-DTM: a case study of ginseng

**DOI:** 10.1186/s13020-017-0148-7

**Published:** 2017-09-12

**Authors:** Qian Chen, Ni Ai, Jie Liao, Xin Shao, Yufeng Liu, Xiaohui Fan

**Affiliations:** 1 School of Pharmaceutical Sciences, Wenzhou Medical University, Wenzhou, 325035 China; 2 Pharmaceutical Informatics Institute, College of Pharmaceutical Sciences, Zhejiang University, Hangzhou, 310058 China

## Abstract

**Background:**

Valuable scientific results on biomedicine are very rich, but they are widely scattered in the literature. Topic modeling enables researchers to discover themes from an unstructured collection of documents without any prior annotations or labels. In this paper, taking ginseng as an example, biological dynamic topic model (Bio-DTM) was proposed to conduct a retrospective study and interpret the temporal evolution of the research of ginseng.

**Methods:**

The system of Bio-DTM mainly includes four components, documents pre-processing, bio-dictionary construction, dynamic topic models, topics analysis and visualization. Scientific articles pertaining to ginseng were retrieved through text mining from PubMed. The bio-dictionary integrates MedTerms medical dictionary, the second edition of side effect resource, a dictionary of biology and HGNC database of human gene names (HGNC). A dynamic topic model, a text mining technique, was used to emphasize on capturing the development trends of topics in a sequentially collected documents. Besides the contents of topics taken on, the evolution of topics was visualized over time using ThemeRiver.

**Results:**

From the topic 9, ginseng was used in dietary supplements and complementary and integrative health practices, and became very popular since the early twentieth century. Topic 6 reminded that the planting of ginseng is a major area of research and symbiosis and allelopathy of ginseng became a research hotspot in 2007. In addition, the Bio-DTM model gave an insight into the main pharmacologic effects of ginseng, such as anti-metabolic disorder effect, cardioprotective effect, anti-cancer effect, hepatoprotective effect, anti-thrombotic effect and neuroprotective effect.

**Conclusion:**

The Bio-DTM model not only discovers what ginseng’s research involving in but also displays how these topics evolving over time. This approach can be applied to the biomedical field to conduct a retrospective study and guide future studies.

**Electronic supplementary material:**

The online version of this article (doi:10.1186/s13020-017-0148-7) contains supplementary material, which is available to authorized users.

## Background

The scientific study of biomedicine is very active but all of the profound results are widely scattered in the literature. The information (Additional file 1: Figure S1), the number of journals and indexed citations in MEDLINE, has been increasing at a considerable rate, so that it becomes a challenging task for researchers to keep up-to-date with relevant scientific information [1, 2]. In response, continuous improvement of PubMed Web service is made by the National Center for Biotechnology Information (NCBI) and many Web tools are developed to fulfill quick and efficient systematic search and retrieve as well as comparable literature search service [3, 4]. Furthermore, great progress has been made in text mining, a technique in conjunction with machine learning and computational statistics [5]. Except several approaches specifically for small and homogenous document collections [6], text mining approaches are designed for rapidly analyzing large quantities of literature to extract meaningful information and yield valuable insights [7, 8]. Some text mining approaches, like CRAB [9], SparkText [8], SWIFT-Review [10] and other topic models [11, 12], have been successfully applied to knowledge discovery in the growing body of literature [1]. Topic modeling, a statistical solution to summarizing large archives of documents, has gained increasing attention in recent years [12]. It enables researchers to discover the themes from an unstructured collection of documents without any prior annotations or labels. latent Dirichlet allocation (LDA), proposed by David M. Blei in 2003, is the most basic topic model [13]. The primary assumptions of LDA are that words in a document are exchangeable and documents exhibit multiple topics. Along with the rapid development of topic modeling in machine learning, there have emerged plenty of exciting extensions of LDA. With the increasing interest on authorship attribution, Michal et al. presented an author-topic model (ATM) which simultaneously focused on the content of documents and the interests of authors [14]. David et al. developed a correlated topic model (CTM) remedying the limitation of LDA without the ability to model topic correlation [15]. When applied to the articles from Science published from 1990 to 1999, the CTM obtained a better fit of the large document collections than LDA [16]. Daniel et al. introduced labeled LDA (L-LDA), a supervised topic model, for addressing the problem of multi-labeled document classification. L-LDA significantly outperformed SVMs on some document collections [17]. Furthermore, many implementations for topic modeling are available. The MATLAB topic modeling toolbox brings topic modeling tools to scientists for free scientific use. Topic models and lda are two R packages for fitting topic models, but they employ different estimation techniques [18, 19].

Latent Dirichlet allocation models and other extensions are successfully used for organization of massive documents, find patterns in a large collections of information and classifying multi-label text. These models assume that the order of documents is of no great importance. The assumption is obviously unrealistic when analyzing long-running collections, such as news, scientific journals or search query commercial textual information. For someone, especially business professionals, scholars and politicians, it is important to keep abreast of tracing the evolution of their related fields, so that they can make correct judgments on some critical problems and take further actions in time. To resolve the problem, several topic models have been developed for fitting how topics evolving over time. Levent et al. proposed a generative model, called segmented author-topic model (S-ATM), for discovering scientific topics and the evolution of topics over time effectively [20]. Online topic model (OLDA) captures not only the thematic patterns but also their changes over time even when a new set of related documents appears [21]. Another LDA-style topic model, topics over time (TOT), can summary a mass of collections and how the structure changes over time with the assumption that topic discovery is determined by both word co-occurrences and temporal information [22]. However, there is another probabilistic time series model, dynamic topic model (DTM), developed for capturing the topic evolution by grouping corpus of documents sequentially. Compared with TOT, DTM fits the models on a series of discretized time slices, for instance by date. The strong assumption of DTM is that topics at current time slice have smoothly evolved from the corresponding topics at previous time slice. It means that the *k*th topic in one epoch only depends on the *k*th topic in its previous epoch. In the field of machine learning, some promising extensions of DTM have been proposed in order to meet different needs [23–25]. These changes improve or extend the methodology. In addition, the presentation of results from topic models has become another promising direction [26]. Some tools have been designed to visualize trend analysis of dynamic topic models [27, 28].

Ginseng, one of famous and precious medicinal materials, has a history of more than 2000 years for human health and medicine in China and Asia. The traditional belief holds that various properties of ginseng, such as tonic effect, can modulate the unbalanced situation of human body. With the addition of significant effects on many situations, ginseng is widely used in traditional Chinese medicine (TCM). According to statistics, at least 3500 TCM formulae contain ginseng, which is the main form of TCM [29]. For example, in *Shanghan Lun*, a classical set of TCM formula which was compiled by a famous doctor Zhang Zhongjing in han dynasty, ginseng is not only one of the well-known herbs, it is used in nearly a fifth of the formulae to benefit many situations. To complement diets or maintain health, ginseng has gained popularity in the West over the last two decades. According to two recent national health interview surveys in the United States, ginseng is widely used as a dietary supplement and is one of the ten most used natural products among both adults and children [30, 31]. Based on the results from PlantLIBRA plant food supplements (PFS) consumer survey, ginseng is the fourth most frequently used botanicals in Germany, Italy, the United Kingdom and six other European countries [32]. Due to the popularity of ginseng and its high efficacy, many new technologies and strategies were applied to studying ginseng and more and more ginseng-related scientific articles are published every year [33–35]. Additional file 1: Figure S2 showed that the number of ginseng-related articles published every year has always been on the rise. As always, there has been a keen interest in ginseng’s research using advanced techniques. Furthermore, retrospective summaries of previous studies has always contribute to great achievements in science. As we mentioned before, topic modeling has been used in medical and biological sciences [36, 37]. To our knowledge, scientific literature is the predominant resource, but has not yet been applied to interpreting the temporal evolution of research domains, like ginseng-related research. The inherent hypothesis of DTM closely matches the formation of scientific research ideas, which proceed gradually in an orderly way. In this paper, taking ginseng as an example, DTM was employed to do a retrospective study and capture the temporal evolution of the research domain of ginseng.

## Methods

### System overview

**Fig. 1:**
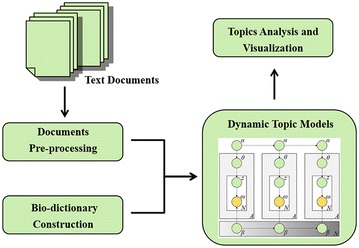
The overview of Bio-DTM. The system consists of four components, documents pre-processing, Bio-dictionary construction, dynamic topic models and topics analysis and visualization

In this paper, a Bio-DTM was established by combining DTM and a bio-dictionary to help researchers mine the related topics how developing over time in a large amount of biomedical documents. Figure 1 illustrates an overview of our Bio-DTM, mainly including four components, documents pre-processing, bio-dictionary construction, dynamic topic models and topics analysis and visualization. Matlab (R2013a) and R (3.1.1) were used to process data in this study. The Additional file 2: Minimum standards of reporting checklist contains details of the experimental design, and statistics, and resources used in this study.

### Documents pre-processing

Documents pre-processing is mainly divided into three steps. The first is to collect the target documents. Then useful information is extracted from the collection of text documents. Lastly, it needs to reduce the noise of the extracted raw data. In the example of this paper, scientific articles pertaining to ginseng were retrieved through text mining with keyword ‘ginseng [Title/Abstract]’ up to the publication date at 2017/7/31 from PubMed. The title, abstract and year of publication were extracted from each scientific article. Then the document-words matrix was generated and then subjected to filtered process. In a raw article, it contains any sort of punctuation and hyphenation. So a bag of words was extracted from the original articles by removing punctuation and stop words. Then stemming process was employed to convert inflected words to their roots so that mapping related words to the same stem as far as possible. It naturally simplifies the feature space through reducing the number of words of each article.

### Bio-dictionary construction

The Bio-DTM in the paper is a dynamic sequential probabilistic model for a text corpus considered as a bag of words. Thus a dictionary of biomedical domain becomes a necessity. The bio-dictionary used in this paper integrates MedTerms medical dictionary, SIDER2, a dictionary of biology and HGNC four resources. MedTerms medical dictionary written by physicians contains a considerable number of classical and contemporary medical terms. SIDER2, the second edition of side effect resource, is used to obtain names of side effects that are extracted from public documents and package inserts. The sixth edition of a dictionary of biology edited by Elizabeth Martin and Robert S. Hine covers primary terms of biology, biophysics and biochemistry. HGNC provides a unique and meaningful names for known human genes. Approved symbol, previous symbols and synonyms of human genes were all included. Considering that the Bio-DTM is based on single word, all collected terms were split apart into individual word. Just like with documents pre-processing, stop words and hyphenations were removed and stemming process was performed to optimize the established Bio-dictionary.

### Dynamic topic models

Latent Dirichlet allocation, introduced by David Blei et al. in 2003, is a generative probabilistic model for text corpora and collections of discrete data. The basic purpose is to facilitate organizing and summarizing information about a large number of electronic files which users are interested in. It’s the most important theoretical concept that each document belongs to a multinomial distribution over topics, and each topic is associated with a multinomial distribution over a set of words which are simply assumed to be exchangeable. At present, it has becoming more and more popular to analyze how topics evolving over time, especially in the fields of academia and business. A dynamic topic model, an extension of the LDA model, emphasizes on capturing the development trends of topics in a sequentially collected documents. In a dynamic topic model, all documents are grouped by time slice and the primary assumption is that topics of this time slice evolve from topics of the previous time slice.

### Topics analysis and visualization

After the topics were obtained via Bio-DTM, the topmost frequent words of each topic were listed. The main research contents of topics were analyzed according to the words with the highest frequency. The evolution of topics was detected based on the probabilities of topics at every time slice. ThemeRiver [28], a prototype system, was employed to visualize the evolution of topics over time within a large collection of documents related to ginseng in this paper.

## Results

### Text documents for DTM

A MEDLINE format result with 5857 ginseng-related articles was downloaded from PubMed by setting the keyword ‘ginseng [Title/Abstract]’ and the publication date from 1975/01/01 to 2017/7/31. But only 5394 articles with available abstract were used for the study. The title, abstract and year of publication of each article were extracted from the MEDLINE result. The main content of per-article is represented by its title and abstract. The number of time-slices depends on the information of publication year. All articles fall into 16 time-slices and the first four time-slices cover all documents published from 1975 to 2005, about 400 articles per slice.

### Bio-dictionary construction

The bio-dictionary used in this paper mainly includes four sources, MedTerms medical dictionary, SIDER2, a dictionary of biology and HGNC. MedTerms medical dictionary contains 16,392 medical terms. SIDER2 records 5373 side effects. A dictionary of biology provides 5492 biological terms. HGNC has 19,094 known human genes. Because of the bag-of-words assumption, all of these sources were concatenated together and were separated into single word. The bio-dictionary ultimately contains 67,696 words by two operations, stemming and removing duplicate.

### Documents pre-processing

The corpus of ginseng in the paper possesses 5394 documents. Every document holds explicit timestamp and textual information consisting of title and abstract. All documents were spilt into 16 groups according to their timestamps, because DTM is an discrete-time model and there is clearly a large discrepancy in the number of ginseng-related literature published per year. Textual information was transformed into a word-frequency matrix. After documents pre-processing, there were only 5384 paper left for analysis. For every document, the DTM format includes the number of unique words qualified in the bio-dictionary, the serial number of words and the number of occurrences of these words. Table 1 shows the number of documents in each time slice.

**Table 1:** The number of documents in each time slice

The serial number of slice	The publication date of paper	The number of paper	The serial number of slice	The publication date of paper	The number of paper
1	1975–1995	389	9	2010	248
2	1996–2000	402	10	2011	343
3	2001–2003	411	11	2012	382
4	2004–2005	340	12	2013	410
5	2006	212	13	2014	426
6	2007	206	14	2015	439
7	2008	210	15	2016	432
8	2009	239	16	2017	295

### Dynamic topic models

A subset of 5384 articles about ginseng from PubMed has been analyzed on a 20-topic dynamic topic model, spilt into 16 time slices between 1975 and 2017. Every topic has changed smoothly across time. For every topic, its topmost frequent words were extracted based on its probability of occurrence in 16 time slices. Table 2 listed 20 discovered topics and its most representative words.

**Table 2:** The topmost frequent words for 20 topics after fitting to the ginseng-related articles by Bio-DTM

Topic ID	Topmost frequent words of topic
1	Renal, genom, amino, biosynthesi, clone, cultivar, yeast, cdna, athlet, polymorph
2	Stem, skin, leaf, bone, dri, phenol, marrow, stage, fibroblast, wound
3	Infect, radic, scaveng, heat, virus, substrat, cyp3a4, influenza, hydroxyl, pgp
4	Diet, muscl, cholesterol, fat, glycosid, adipocyt, ppargamma, antibodi, ampk, insulin
5	Fraction, polysaccharid, ferment, pesticid, residu, column, frg, transit, neutral, pectin
6	Strain, soil, genus, nov, warfarin, polar, bacterium, minor, dsm, genom
7	Cam, tcm, irradi, hair, radiat, sperm, exhaust, alkalin, train, contamin
8	Channel, ca2, cardiac, relax, oocyt, contract, muscl, eno, lpa, ion
9	Injuri, women, fatigu, myocardi, estrogen, ischemia, menopaus, syndrom, cerebr, ischem
10	Pharmacokinet, ion, formula, prescript, ppd, rhizom, ppt, urin, raw, excret
11	Cultiv, temperatur, wild, seed, white, embryo, genet, flower, somat, pathogen
12	Cancer, lung, colon, intak, metastasi, colorect, ventricular, pulmonari, failur, mmp9
13	Diabet, intestin, insulin, toler, digoxin, absorpt, pancreat, beta, grg1, morphin
14	Apoptosi, macrophag, nfkappab, ros, cox2, cancer, mitochondri, angiogenesi, arrest, p38
15	Transform, medium, hairi, transgen, cold, degrad, spectroscopi, biomass, ml1, callus
16	Breast, vaccin, adjuv, discrimin, prostat, skin, fingerprint, antibodi, metabolom, antigen
17	Pressur, hepat, oil, ethanol, metal, aqueous, allerg, hepatotox, fibrosi, asthma
18	Platelet, lymphocyt, spleen, aggreg, pain, cisplatin, nrf2, antiplatelet, milk, camp
19	Berri, behavior, alcohol, depress, memori, swim, ach, learn, avoid, erectil
20	Neuron, cognit, memori, hippocampus, drink, behavior, glutam, energi, astrocyt, task

### Topics visualization

**Fig. 2:**
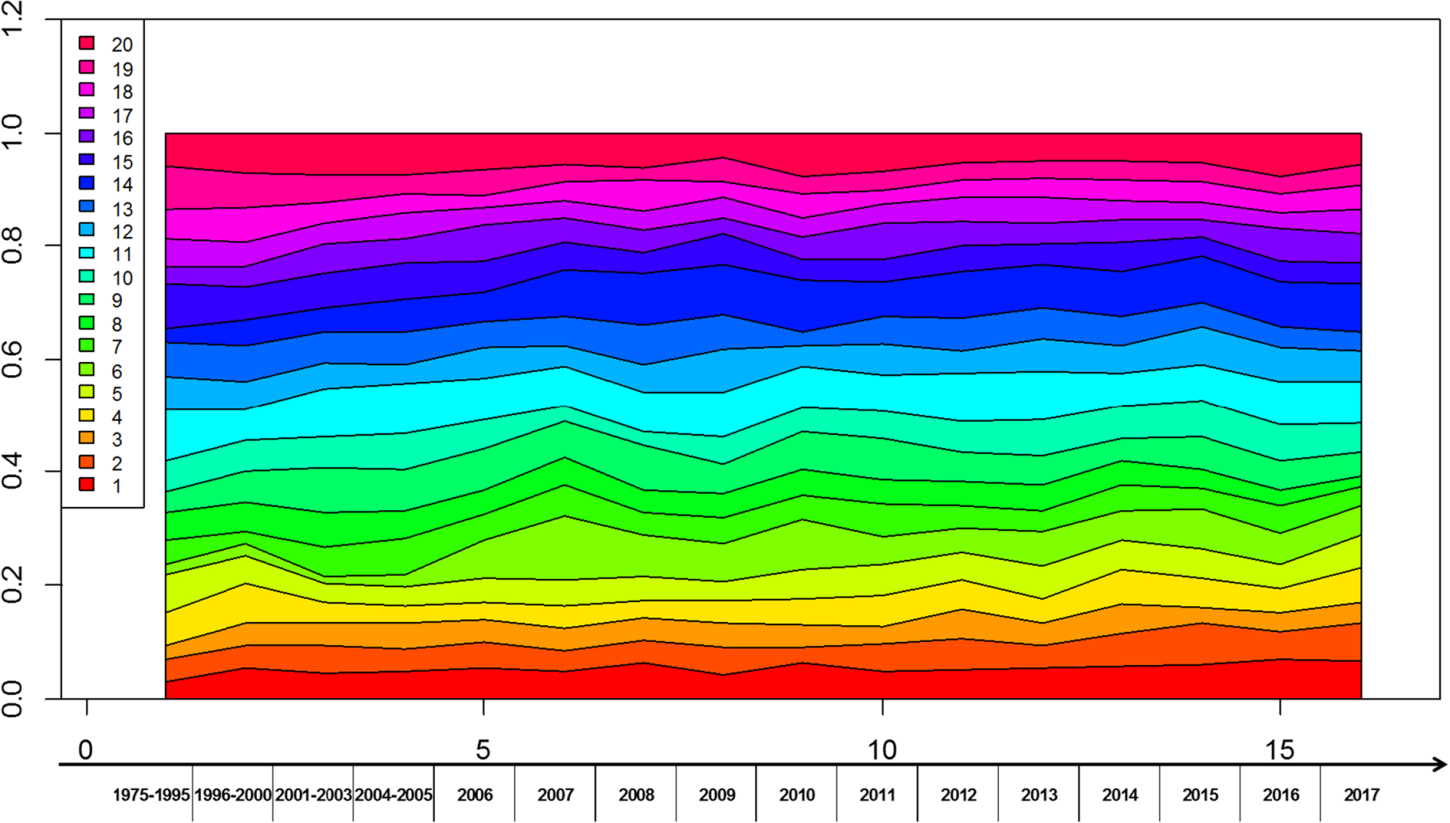
Visualization of ginseng-related topics discovered by Bio-DTM. The river stands for the 20 topics and their evolution over 16 time slices. The river, horizontally flowing from left to right, represents the whole collection time shaft from 1975 to 2017 and different colored currents represent the 20 topics, respectively. The width variation of every river visually depicts changes in strength or popularity of the corresponding topic over time slice

Figure 2 illustrates the evolution of 20 topics from 1975 to 2017 discovered from a large collection of ginseng-related articles. The river, horizontally flowing from left to right, represents the whole collection time shaft and colored currents represent topics. The changing width of a river visually depicts transient changes in strength or popularity of the corresponding topic.

## Discussion

In this paper, we developed a Bio-DTM model by extending the DTM model to reveal topics and their evolution in biomedical literature. PubMed, accessing primarily the MEDLINE database, provides abundant biomedical literature for free. MEDLINE contains over 5600 biomedical journals from all around the world and the abstract coverage dates back to 1946. The Bio-DTM model in this paper emphasizes on bio-terms occurred in biomedical literature. A restricted bio-vocabulary was used as input parameter to measure their distribution on topics. The bio-vocabulary integrates MedTerms medical dictionary, SIDER2, a dictionary of biology and HGNC four databases, including genes, side effects, diseases and so on. Finally, 5384 articles with available abstract and a bio-dictionary covering 67,696 words are available for Bio-DTM model. All documents were spilt into 16 groups according to their timestamps and textual information was transformed into a word-frequency matrix. Textual information, consisting of title and abstract, was optimized by two restricted restrictions. One is the total frequency of words more than once. The second is that only words with a term frequency-inverse document frequency (tf-idf) a bit more than the first quartile were included. Tf-idf reflects how important a word is to a document in a corpus by a method of numerical statistic. So the second restriction allows to elide words which have low frequency as well as those occurring in many documents.

Twenty topics have been discovered from the large collection of ginseng-related articles and the most representative words of every topic were list in Table 2. Topic 1 focuses on the landscape, such as ginsenoside biosynthesis, physiological traits, polymorphism, of ginseng or the differences among ginseng cultivars by genomic and transcriptomic studies. Topic 6 reminded that the cultivation of ginseng is one of the major research fields of ginseng. The research contents of topic 9 mainly involved in the application of ginseng in dietary supplements, complementary and integrative health practices. Based on the content of topic 10, it is primarily on pharmacokinetics of ginseng and Chinese herbal formulae containing ginseng. Besides, the Bio-DTM model gave us insight into the main pharmacologic effects of ginseng, like anti-aging effect (topic 2), immunomodulatory activity (topic 3), anti-metabolic disorder effect (topic 4), cardioprotective effect (topic 8), anti-cancer effect (topic 12), anti-diabetic activity (topic 13), anti-inflammatory and anti-apoptosis activities (topic 14), hepatoprotective effect (topic 17), anti-thrombotic effect (topic 18), anti-depressive effect (topic 19) and neuroprotective effect (Topic 20).

To better display how topics change over time, ThemeRiver, a prototype system, was employed to visualize topic variations over time. Figure 2 illustrates the evolution of 20 topics from 1975 to 2017 discovered from a large collection of ginseng-related articles. The river horizontally flows from left to right, which represents the whole collection time shaft. Each colored current represents one topic. The changing width of a river visually depicts changes in strength or popularity of the corresponding topic.

**Fig. 3:**
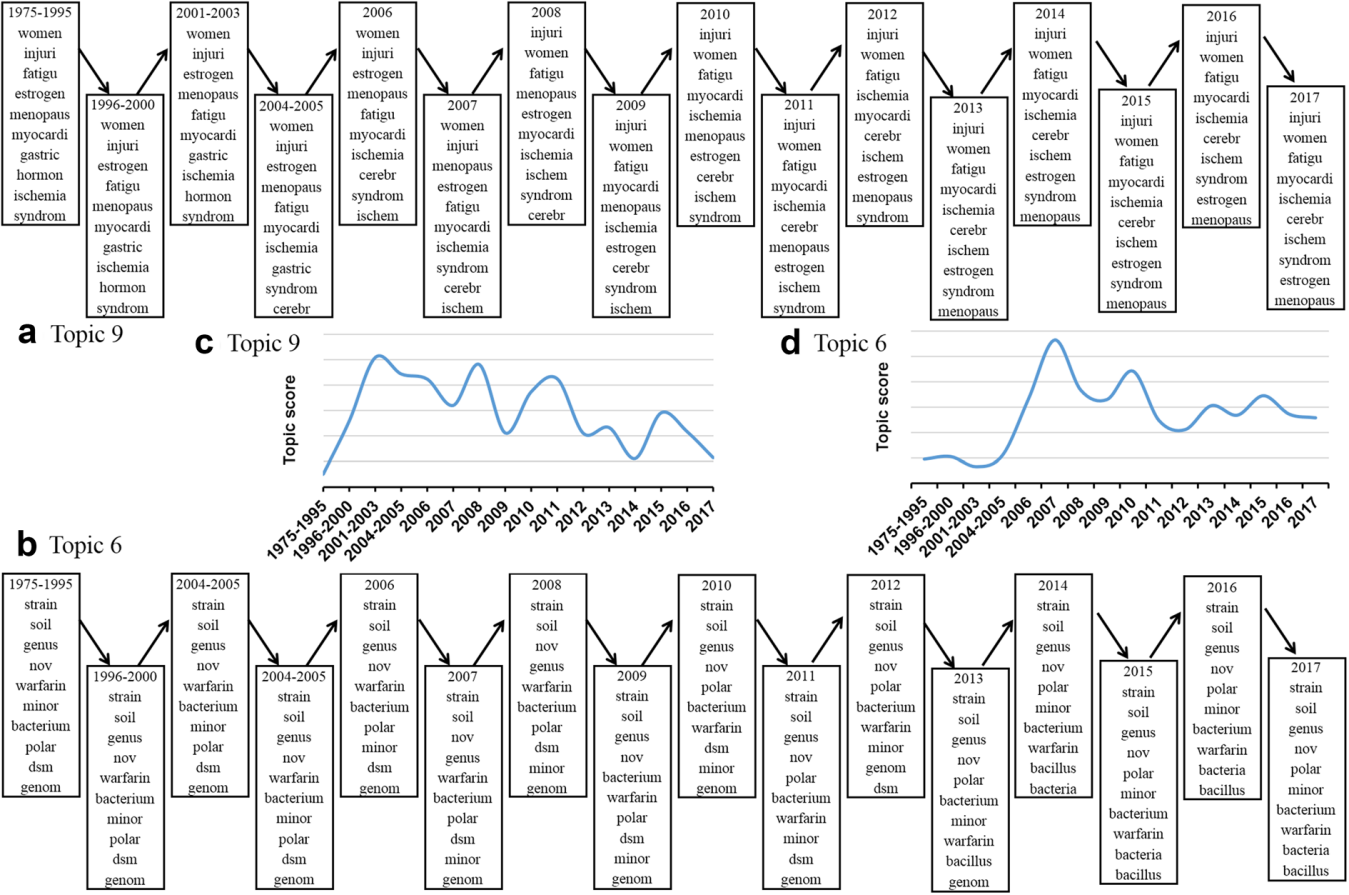
Topics from biological dynamic topic model on ginseng-related literature. The top 10 words at each time slice have been illustrated for topic 9 (**a**) and topic 6 (**b**); the topic score reflects how its topic has changed over time with **c** for topic 9 and **d** for topic 6

The width variation of each current reflects the matching topic strength at each time slice. For example, in Fig. 2 the topic 9 increases the relative popularity in the early twentieth century as indicated by broadening the spring green current. As shown in Fig. 3a, c, topic 9 showed that ginseng was used for dietary supplements, complementary and integrative health practices. Under the condition of legislative favors, in 1999, the National Center for Complementary and Alternative Medicine (NCCAM), a division of the National Institutes of Health (NIH), had been established to explore complementary and alternative healing practices in the context of rigorous science. According to NIH state-of-the-science conference statement on management of menopause-related symptoms in 2005, ginseng may be favorable for some menopausal symptoms, such as daily mood symptoms and sleep disturbances, and with one’s overall sense of well-being [38]. According to the 2002 National Health Interview Survey, 19% of adults took natural products and ginseng was the second most commonly used natural products [39]. While in 2007, 17.7% of American adults took natural products and ginseng came in fifth follow after fish oil/omega 3/DHA, glucosamine, echinacea and flaxseed oil or pills [40]. Moreover, in the light of nutraceutical guidelines for endocrine practice in 2003, ginseng is one of dietary supplements and nutraceuticals used in functional medicine for stress and menopause conditions [41]. These all contribute to the popularity of topic 9 in the beginning of the twentieth century.

From Fig. 3b, d, it is obvious that topic 6 mainly has with respect to the planting of ginseng, especially symbiosis and allelopathy. The width variation of this topic in the Fig. 2 reflect that it has become stronger since the mid-twentieth century. In the past for a long time, people had to cut forest to cultivate ginseng because of the unable successively cultivation. Aiming at improving ecological conditions, the Chinese government initiated the Natural Forest Protection Program and the Slope Land Conversion Program in the twentieth century [42]. The ecological rehabilitation projects not only protect existing natural forests from excessive cutting but also plan to restore natural forests in particular in ecologically sensitive areas [43]. Therefore, research of growing and cultivation on ginseng should be enhanced and it needs to make the fullest use of each part of the plant, such as leaf, flower and stem [44, 45]. To obtain high-quality ginseng and improve the productivity of ginseng, the research highlights have been turned to endophytic microorganisms and allelopathy [46, 47]. There are many reports on endophytes in ginseng and their roles, such as plant growth promotion and antifungal activity [48, 49]. As shown in Fig. 3d, the content of topic 6 became a research hotspot in 2007.

## Conclusion

In this paper, we have employed the Bio-DTM to model the evolution of ginseng-related topics. The main advantage is that the inherent hypothesis of DTM, that topics supposed to evolve smoothly from their previous state, closely matches the formation of scientific research ideas. The DTM model not only discovers what ginseng’s research is involving in but also displays how these topics have evolved over time. This approach is well-suited for conducting a retrospective study as well as has a wide application in biomedical field.

## Additional files

**Additional file 1: Figure S1.** Growth of MEDLINE. The number of journals in MEDLINE and the number of indexed citations added to MEDLINE during each fiscal year from 2000 to 2012. The data was from MEDLINE® STATISTICS in the Official Website ( http://www.nlm.nih.gov/bsd/pmresources.html). **Figure S2.** The trend of ginseng-related articles that published in PubMed from 1975 to 2016.

**Additional file 2.** Minimum standards of reporting checklist.

**Additional file 3.** Ginseng-related articles for DTM.

## Abbreviations

DTM: dynamic topic model
LDA: latent Dirichlet allocation
TOT: topics over time
tf–idf: term frequency–inverse document frequency
L-LDA: labeled latent Dirichlet allocation
CTM: correlated topic model
