# Supplementary material for: Revealing topics and their evolution in biomedical literature using Bio-DTM: a case study of ginseng
Source: Chin Med. 2017 Sep 12;12:27. doi: 10.1186/s13020-017-0148-7 (PMC5596940; doi:10.1186/s13020-017-0148-7)
Supplement: Supplementary file 1 — Additional file 1: Figure S1. Growth of MEDLINE. The number of journals in MEDLINE and the number of indexed citations added to MEDLINE during each fiscal year from 2000 to 2012. The data was from MEDLINE® STATISTICS in the Official Website ( http://www.nlm.nih.gov/bsd/pmresources.html). Figure S2. The trend of ginseng-related articles that published in PubMed from 1975 to 2016. [file 13020_2017_148_MOESM1_ESM.doc]

***Supplementary Material for:***

Revealing Topics and their Evolution in Biomedical Literature Using Bio-DTM: A Case Study of Ginseng

*Qian Chen*^1,2^*,* *Ni Ai*^2^*, Jie Liao*^2^*, Xin Shao*^2^*,* *Yufeng Liu*^2^ *and Xiaohui Fan*^2^***

^1^ *School of Pharmaceutical Sciences, Wenzhou Medical University, Wenzhou 325035, China;*

^2^ *Pharmaceutical Informatics Institute, College of Pharmaceutical Sciences, Zhejiang*

*University, Hangzhou 310058, China.*

*Email addresses of authors:*

*11319032@zju.edu.cn (Qian Chen); 1410070533@qq.com (Ni Ai); 493298159@qq.com (Jie Liao); 610461928@qq.com (Xin Shao); 1436346597@qq.com (Yufeng Liu).*

** Author to whom correspondence should be addressed; E-Mail: fanxh@zju.edu.cn;*

*Tel.: +86-571-88208596; Fax: +86-571-88208426.*


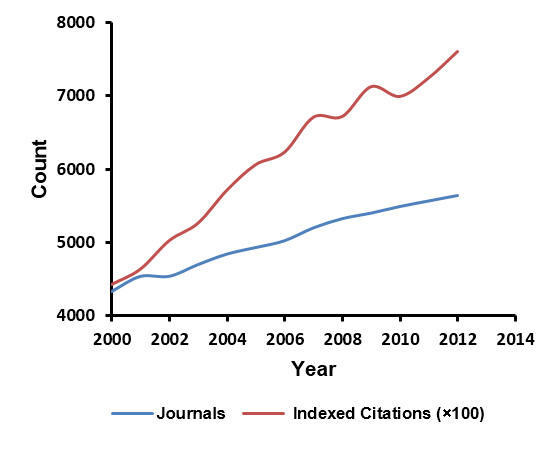


Figure S1. Growth of MEDLINE. The number of journals in MEDLINE and the number of indexed citations added to MEDLINE during each fiscal year from 2000 to 2012. The data was from MEDLINE® STATISTICS in the Official Website ( <http://www.nlm.nih.gov/bsd/pmresources.html> ).


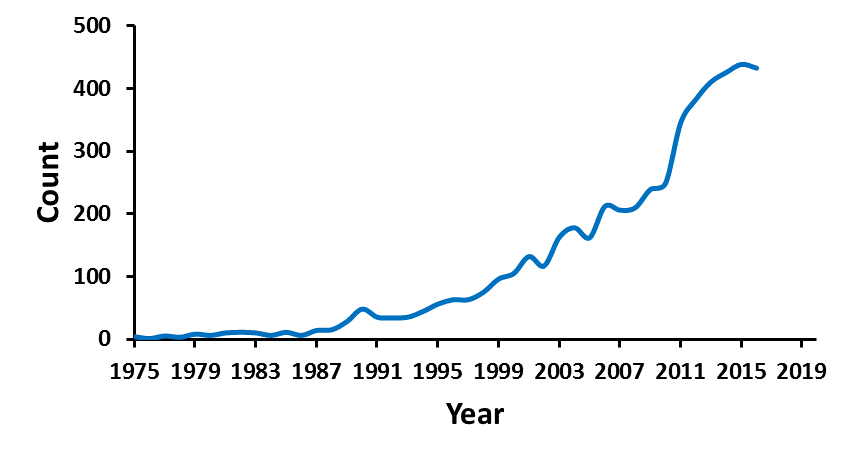


Figure S2. The trend of ginseng-related articles that published in PubMed from 1975 to 2016.
